# The influence of ceramic tile characteristics on visual comfort and cognitive performance in workplace environments: a study based on VR and EEG

**DOI:** 10.3389/fpsyg.2026.1639942

**Published:** 2026-03-17

**Authors:** Jiayin Chen, Yue Cheng, Qingyun Guo, Feng Li, Jue Wu, Ming Yang, Erping Xiang

**Affiliations:** 1 School of Art and Design, Zhejiang Sci-Tech University, Hangzhou, China; 2 College of Architecture and Urban Planning, Tongji University, Shanghai, China; 3 Art and Design College, Tianjin University of Science and Technology, Tianjin, China; 4 School of Fashion Design and Engineering, Zhejiang University of Science and Technology, Hangzhou, China; 5 Academy of Fine Arts, Jishou University, Jishou, China

**Keywords:** ceramic tiles, cognitive performance, EEG, visual comfort, workplace environment

## Abstract

**Background:**

Ceramic tiles are widely used in workplace environments, and their visual characteristics can influence users’ perceptual and cognitive experiences. However, research remains limited on how ceramic tile properties affect visual comfort, cognitive performance, and related neural responses.

**Method:**

This study examines six common types of mass-produced ceramic tiles, employing virtual reality (VR) and electroencephalogram (EEG) technologies to evaluate visual comfort and cognitive performance. A 2 (patterned vs. non-patterned) × 3 (light-toned, medium-toned, dark-toned) factorial design was used to construct six immersive VR environments through 3D modeling. Data on visual comfort ratings, cognitive behavioral performance, and EEG responses were collected from participants in each environment. The effects of tile’s pattern and brightness on visual comfort, cognitive outcomes, and neural activity were analyzed.

**Result:**

The findings indicate that environments with non-patterned and light-toned ceramic tiles received higher visual comfort ratings and elicited increased alpha wave power spectral density, reflecting positive emotional experiences. In terms of cognitive performance, the participants had shorter reaction durations in the environments of light-toned ceramic tiles and patterned ceramic tiles, and were negatively correlated with the beta wave power spectral density values.

**Discussion:**

The results suggest that ceramic tile selection can be tailored to the functional needs of interior spaces. For instance, in relaxing settings such as lounges, tiles that enhance alpha-wave activity may be preferable, while in workspaces requiring high cognitive engagement--such as studios, meeting rooms, or offices--tiles that promote moderate beta-wave activity may be more suitable. This study provides valuable insights and evaluation methods for interior and ceramic tile designers, especially for design of Chinese region.

## 1 Introduction

### 1.1 Research background

In modern society, most individuals work indoors, and the quality of indoor workplace environments significantly influences human well-being and performance (Clements-Croome, 2006; Al horr et al., 2016). Ceramic tiles are widely used in interior workplace decoration across Asia, Europe, the Americas and the Middle East due to their esthetic appeal and ease of maintenance (Van Lemmen, 2013). According to Chu (2021), ceramic tiles account for 71.4% of indoor floor materials in China, indicating their extensive application. Liu (2025) further highlighted the high consumption of ceramic tiles in countries including China, Brazil, Spain, Portugal, and Malaysia.

In addition to consumer preferences, cross-national differences in ceramic tile application are closely associated with cultural traditions. For instance, China has a long history of using brick materials for flooring since the Spring and Autumn and Warring States Periods (Knapp, 2012). The cultural perception that brick-based materials convey cleanliness and stability, together with the practical advantages of ceramic tiles (Yu and Wang, 2010), has promoted their popularization in China. Even in the United States, ceramic tiles account for approximately 30% of flooring materials (FCW, 2024).

Individuals primarily perceive tile characteristics through vision, which is the dominant sensory pathway (Artacho et al., 2020). As a key interior design element, tiles frequently enter people’s sight and may influence visual comfort as well as cognitive responses. However, research on the effects of tile characteristics on visual comfort and cognitive performance remains limited. Considering the wide use of tiles in offices, classrooms and other cognitively demanding spaces, it is essential to investigate how their visual characteristics objectively influence cognitive performance and visual comfort.

In recent years, researchers have examined human perception and cognitive performance related to interior elements using behavioral and neural measures (St-Jean et al., 2022). Such studies are commonly supported by Evidence-Based Design (EBD), which applies scientific approaches to evaluate environmental impacts on health and efficiency (Bower et al., 2019; Ulrich et al., 2008; Iyendo et al., 2016). Previous EBD research has indicated that interior features influence comfort and cognitive performance (van den Berg et al., 2017; Li J. et al., 2021), and EEG has been used to explore related brain mechanisms (Klimesch, 1999). Thus, EBD studies often integrate subjective, behavioral, and physiological indicators (Ke et al., 2021; Cheng et al., 2024; Sun et al., 2024). Based on this framework, this study investigates how ceramic tiles affect human perception using physiological and behavioral data.

### 1.2 Literature review

#### 1.2.1 Visual comfort research on ceramic tiles

In studies on the visual comfort of ceramic tiles, existing research has primarily relied on questionnaires to assess subjective evaluations, including satisfaction, comfort, emotional valence, and arousal. For example, Serra et al. (2022) found that lighter or monochromatic wall and floor colors significantly enhance individuals’ subjective satisfaction with the environment. Similarly, Mahmood and Tayib (2021) reported that esthetically pleasing tiles in interior design can improve users’ psychological comfort. In contrast, relatively few studies have employed physiological measurement methods. Among those that have, researchers typically assess visual comfort by monitoring participants’ skin conductance and event-related potentials (ERPs) while they view images of ceramic tiles. In two of our previous studies, ERP technology was used to investigate esthetic preferences and neural responses during first impressions of tile photographs within the first second of exposure (Chen and Cheng, 2022; Chen et al., 2023). These studies revealed correlations between tile’s pattern, color tones, and specific ERP components related to esthetic preference. However, esthetic preference represents only one dimension of visual comfort. Laparra-Hernández et al. (2009) found that changes in tile design significantly affected participants’ tension levels, as measured by skin conductance, but did not significantly influence their pleasure. Conversely, Agost and Vergara (2014) observed that tile design features can influence emotional valence. Light-toned tiles, for example, were more likely to elicit feelings of pleasure. Their study also indicated that design features could evoke associations with cleanliness, brightness, and expense, as well as emotional states such as pleasure, calmness, and tension. Notably, these studies primarily involved participants viewing tile images for short durations. However, in real-world settings, visual interaction with ceramic tiles tends to be three-dimensional and sustained over longer periods. Therefore, further investigation is needed to explore the visual comfort of interior ceramic tiles in more immersive and temporally extended contexts.

#### 1.2.2 Application of VR technology in environmental perception research

VR technology offers a high degree of realism and enhanced experimental control and feasibility (Rebelo et al., 2012). Previous studies have employed simulated environments, VR environments, and real-world settings to investigate environmental experiences. Among simulated environments, climate laboratories represent a more advanced type, emphasizing variables such as spatial scale, lighting, temperature, and humidity (Clements et al., 2019). However, the high cost and substantial spatial requirements of climate labs present practical limitations. With continuous improvements in VR technology and increased fidelity in 3D modeling, VR has become an increasingly popular tool for studying environmental perception. Its applications now span diverse fields such as gaming, education, tourism, and architecture (Tian et al., 2021). In recent years, researchers in architecture and environmental design have widely adopted VR to investigate user experiences. For example, Taehoon et al. (2019) found that virtual environments can closely replicate real-world settings, reporting no significant differences in participants’ visual comfort and satisfaction between virtual and physical spaces. Accordingly, the present study uses VR environments to examine perceptual and neural responses to indoor settings featuring different types of ceramic tiles.

#### 1.2.3 Effects of indoor environmental visual features on cognitive performance

In addition to visual comfort, cognitive performance is also an important aspect of the impact brought by the visual characteristics of tiles. Previous research has shown that the visual features of an environment can affect attention levels, cognitive load, and emotional states, thereby impacting cognitive performance (Choi et al., 2014; Plass and Kalyuga, 2019). Evidence-based improvements in indoor environmental design could enhance cognitive performance and associated behaviors. To optimize workplace efficiency, it is essential to consider visual characteristics alongside other environmental factors such as noise, temperature, and air quality (Li J. et al., 2021). Numerous studies in classroom and workplace settings have examined variables such as spatial layout, color, and lighting, confirming their significant influence on cognitive outcomes. For example, Li K. et al. (2021) reported that university students exhibited higher attention and work efficiency in spaces containing large-leafed plants compared to those with small-leafed plants. Lu et al. (2020) found reading efficiency significantly improved under neutral color-temperature lighting. Additionally, Li J. et al. (2021) noted higher cognitive task accuracy in rooms featuring green plant walls and ordinary white walls compared to those decorated with red or blue walls. Ceramic tiles frequently cover extensive areas of indoor surfaces, such as floors and walls, making it crucial to empirically investigate their effects on cognition. Examining reaction time and accuracy in tile-covered environments can provide designers and researchers with valuable insights into cognitive performance, facilitating informed decisions to enhance workplace efficiency through improved environmental attributes.

#### 1.2.4 Evaluation methods of cognitive performance

Selective attention and working memory are important manifestations of people’s cognitive performance at work (Wang X. et al., 2019; Wang S. Y. et al., 2019). The evaluation of environmental impacts on cognitive performance can be conducted through cognitive tasks such as numerical calculations, Stroop tests, and digit span tasks (Wang et al., 2021). Selective attention refers to the process by which individuals consciously use cognitive resources to focus on a chosen stimulus while avoiding irrelevant perceptual inputs that may cause confusion (Kakizaki, 1984). It is typically assessed through tasks involving numerical calculations, logical reasoning, and visual searches. Impulse control is the ability to stop executing overlearned and dominant responses in conflict tasks (Dallaway et al., 2023). It can be measured using Stroop tests. Working memory research typically examines the ability to remember information over short delays (Engle, 2002), commonly evaluated through forward digit-span tasks or more complex backward digit-span tasks. Cognitive task performance is usually measured in terms of response time and accuracy. Employing diverse cognitive experiments (numerical calculation, Stroop, and DST) allows for a comprehensive understanding of cognitive performance within ceramic tile environments, which can help researchers investigate how these environments affect cognitive activities and task execution.

### 1.3 Research gap

Existing research on ceramic tile visual comfort is largely confined to subjective questionnaires or short-term physiological measurements using static images. While some studies have used event-related potentials (ERPs) or skin conductance, they focus on short-term esthetic preferences or emotional reactions, neglecting immersive, long-term scenarios. Traditional experimental paradigms (e.g., climate laboratories) are cost- and space-constrained, and virtual reality (VR)-based studies on ceramic tile environments remain scarce.

Furthermore, current scholarship inadequately addresses the impact of ceramic tile visual features on occupants’ objective cognitive performance (e.g., selective attention, impulse control, working memory). Empirical evidence linking tile design to cognitive outcomes (response time, accuracy) remains insufficient, despite tiles’ widespread indoor application and implications for environmental optimization.

To fill these gaps, this study constructs highly immersive VR indoor environments with different tiles, integrating ERP recordings and cognitive tasks (numerical calculation, Stroop test, digit span task) to explore visual comfort and cognitive performance, providing valid evidence for interior tile design.

### 1.4 Research purpose

In previous study (Chen et al., 2024), we established a method for evaluating the visual comfort of ceramic tiles. Questionnaires can effectively assess visual comfort in situations where physiological measurement techniques are unavailable. This paper applies electroencephalogram (EEG) and VR technologies to further refine the physiological measurement methods for assessing the visual comfort of tile designs from the perspective of neural responses. Additionally, the visual characteristics of the environment may influence cognitive performance. Therefore, the cognitive performance assessment methods in this study leverage VR technology, allowing participants to perform a series of cognitive tasks (Numerical calculations, Stroop, and memory tests) in a VR environment. By collecting data on cognitive performance and neural responses through cognitive behavior experiments, we aim to establish a cognitive performance assessment method related to the design of indoor ceramic tiles.

The results of this study can provide evaluation methods and practical guidance for environmental design in workplaces involving ceramic tiles. To achieve this objective, this study addresses the following research questions:

1. In working environments decorated with tiles featuring different characteristics, are there significant differences in visual comfort ratings and associated neural responses?
2. When performing cognitive tasks in workplace environments featuring tiles with different visual attributes, are there significant differences in cognitive performance and related neural responses?

## 2 Materials and methods

### 2.1 Materials

**Figure 1:**
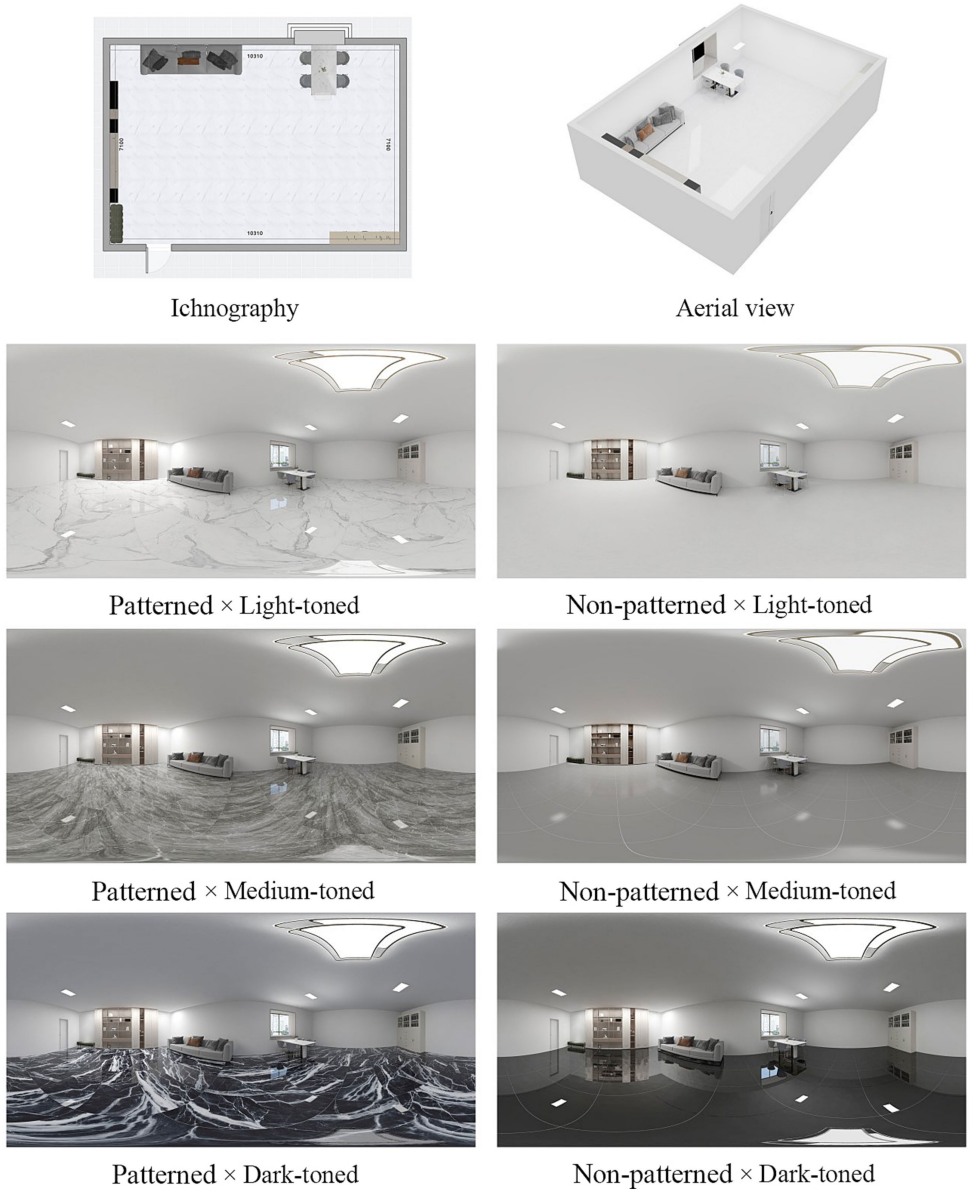
VR environments featuring different ceramic tile designs.

This study employs VR environments featuring different interior ceramic tiles, created using 3D modeling software, as experimental stimuli. When constructing the VR environment through 3D modeling, except for the tile pattern (with/without marble pattern) and brightness (light/medium/dark), other design elements (wall color, furniture style and layout, window frame color) were maintained in a neutral and coordinated configuration, complying with office space design codes to avoid the interference of compositional imbalance on experimental results. The background environment of the experiment adopted an typical layout of ordinary office commonly found in Chinese office buildings. To mitigate participant fatigue from repeated exposure and considering the duration of VR experiments as well as the generalizability of the findings, this study focuses on two variables: tile’s pattern and brightness. Based on previous research (Chen et al., 2024) and market surveys, marble-patterned tiles are the most common type of tile patterns. Therefore, in this study, the pattern level of the tiles was set as those with an imitation marble pattern (patterned tiles) and those without such a pattern (non-patterned tiles). To systematically compare the impacts of various ceramic tiles on visual comfort and cognitive performance, the tiles were categorized into six versions (2 pattern conditions × 3 brightness levels) without altering other interior design elements, as illustrated in Figure 1. The ceramic tiles selected in this paper are mainstream types screened by the authors based on a market survey of China’s ceramic tile industry (Chen et al., 2024). Their friction coefficient (≥ 0.5) complies with the regulatory requirements for dry indoor environments (China Building and Sanitary Ceramic Standardization Technical Committee (SAC/TC 249), 2015). Since the selected tiles are all popular styles from China and the office layout follows the typical pattern commonly seen in ordinary Chinese office buildings, the materials used in this experiment may better align with the preferences of the Chinese population for office decoration. After selecting the ceramic tile flooring for the experiment, the furniture and indoor lighting levels were uniformly set. Controlling the environmental factors other than the tiles does not mean separating or simplifying the design. It is to obtain a more accurate result of the influence of this factor. This research method of testing a single element has been confirmed in many previous authoritative studies (Ulrich, 1984; Li J. et al., 2021; Agost and Vergara, 2014; Choi et al., 2015; Cruz-Garza et al., 2022). Participants viewed each environment from a fixed panoramic perspective situated centrally within the room. Subsequently, these six environments were rendered as 360-degree panoramic images and imported into Unity software. Additionally, all cognitive task stimuli and visual comfort questionnaires were integrated into Unity. To facilitate cognitive task assessments within the VR environment, task stimuli were displayed on the walls of each virtual scene, simulating indoor projections.

### 2.2 Cognitive task design

**Figure 2:**
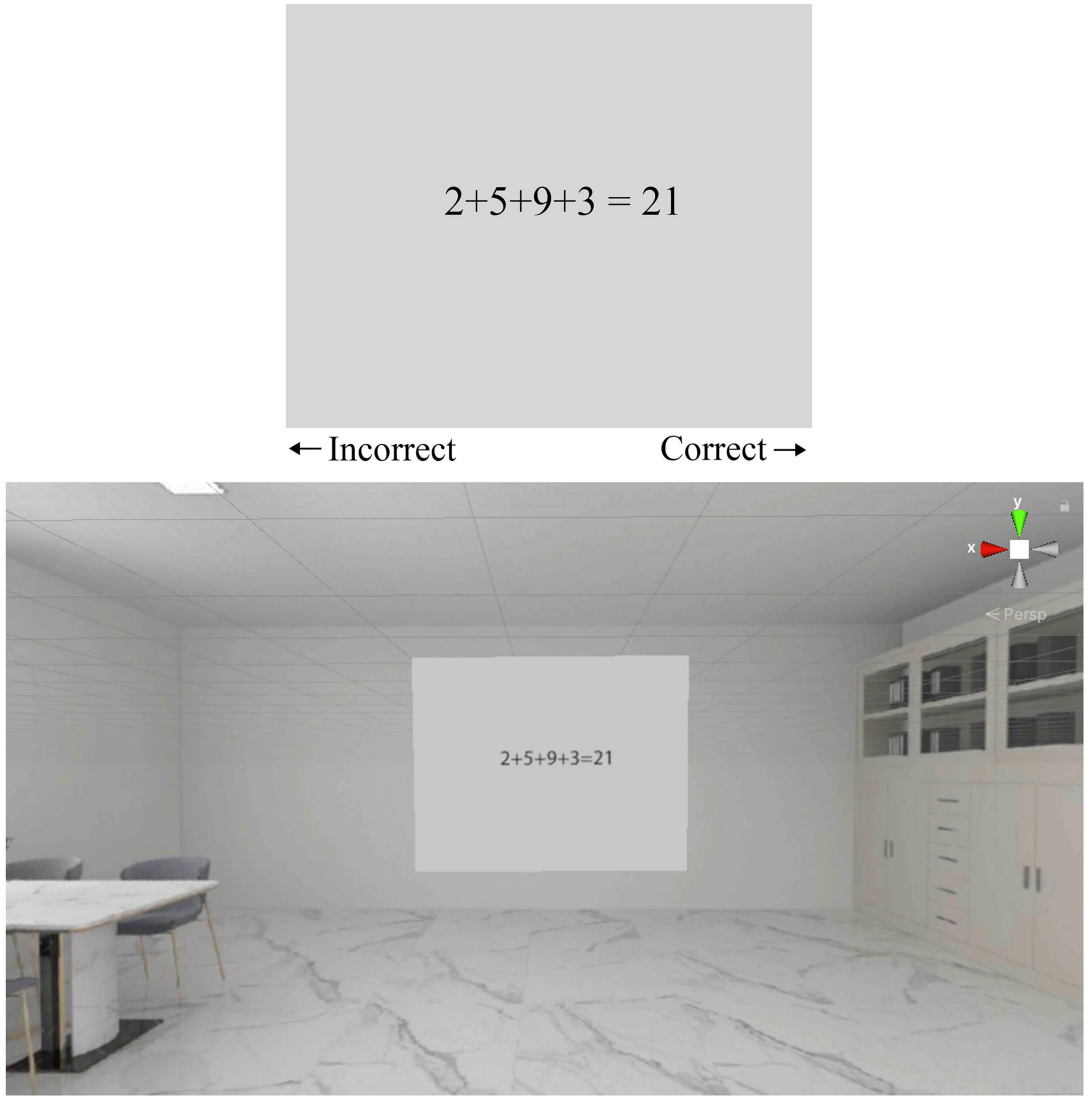
Digital arithmetic task interface.

Considering that people’s work often involves calculation, discrimination and memory, this experiment designed three types of cognitive tasks—numerical calculation, Stroop word-color interference, and backward digit span. In the numerical calculation task, cognitive performance was assessed through simple addition problems. Participants were instructed to press the right mouse button if the result displayed in the VR projection area was correct, and the left mouse button if it was incorrect. Figure 2 illustrates the numerical calculation task within the VR environment, using patterned, light-toned ceramic tiles as an example.

**Figure 3:**
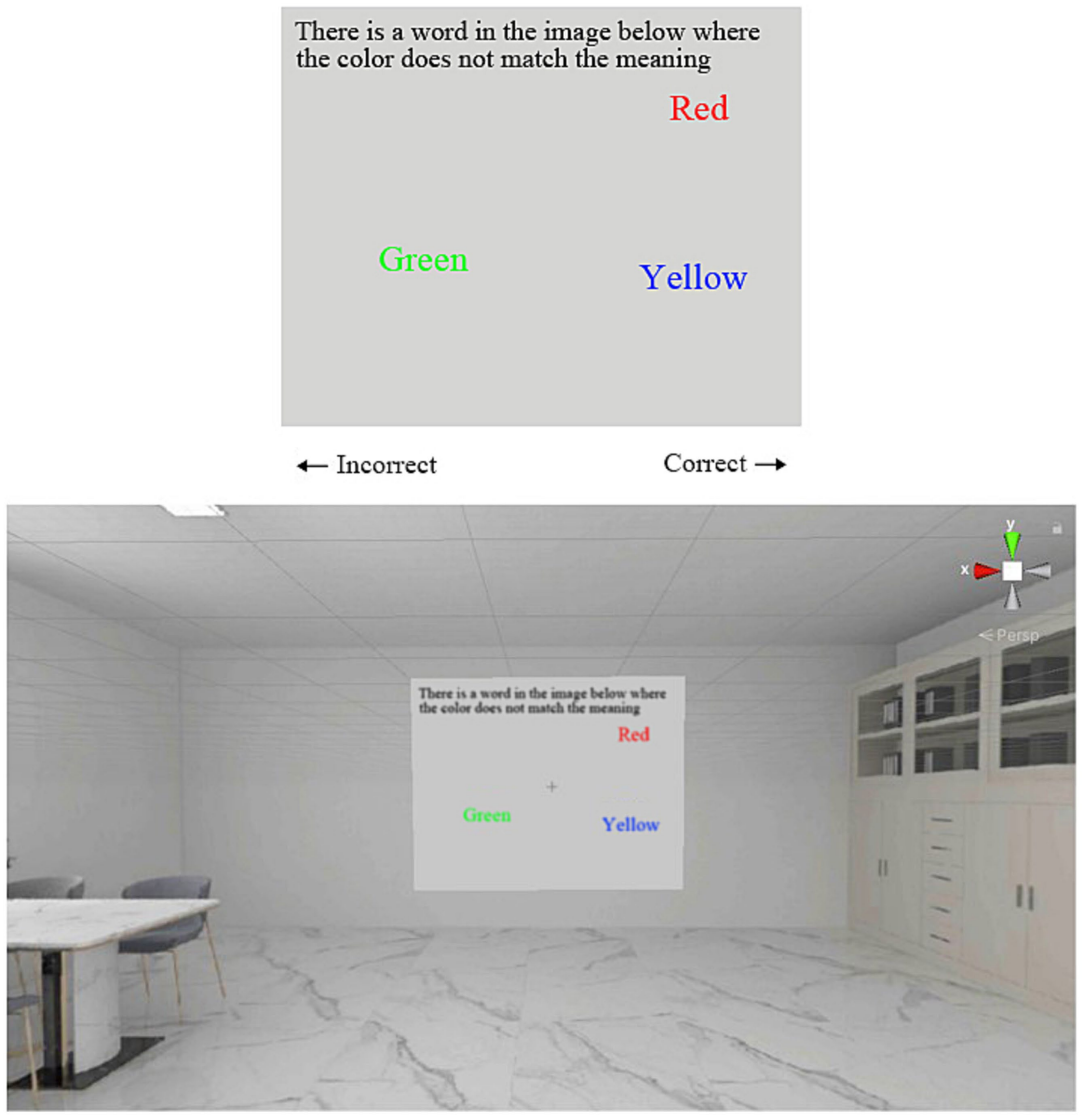
Stroop task: word-color interference paradigm.

The Stroop word-color interference test (Chen et al., 2008) assessed impulse control by requiring participants to identify mismatches between the semantic meaning of words and their displayed colors. Figure 3 demonstrates the Stroop task within the VR environment, using patterned, light-toned ceramic tiles as an example. A text description in the upper-left corner incorrectly states there is one mismatch between word and color, though two mismatches exist. Participants pressed the right mouse button if the description was correct and the left mouse button otherwise.

**Figure 4:**
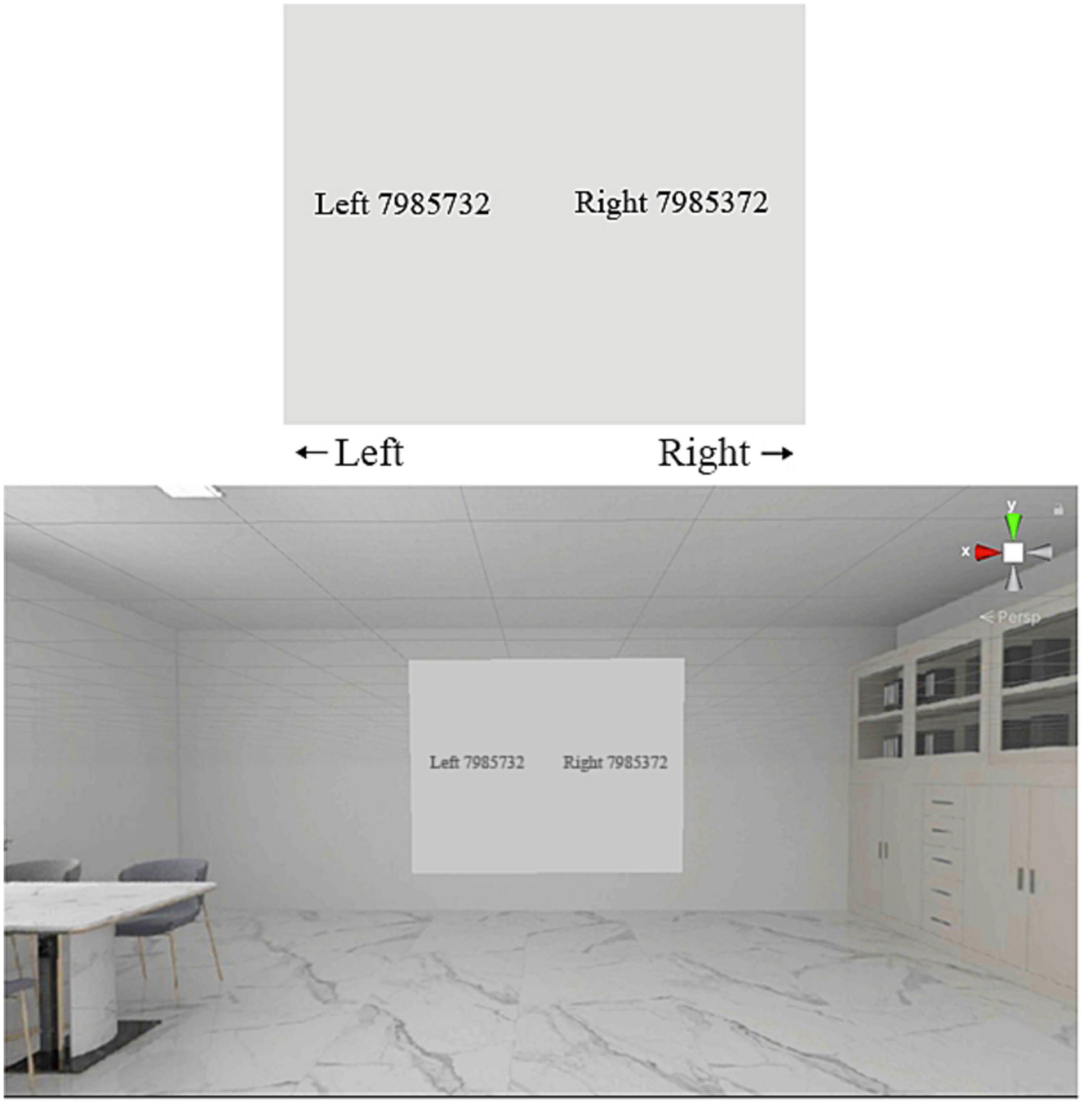
DST for working memory assessment.

Working memory was assessed using the Digit Span Test (DST; Groth-Marnat and Baker, 2003; Wei and Yan, 2008), which evaluates attention, encoding, and auditory processing. During the DST, sequences of 4 to 7 digits were read aloud, after which two digit sets were displayed in the virtual environment. Participants selected the correct set matching the previously heard digits by pressing the corresponding mouse button. Figure 4 illustrates the working memory task within the VR environment, again exemplified by patterned, light-toned ceramic tiles.

To avoid differences in the difficulty levels of cognitive tasks across various scenes, the authors conducted multiple preliminary experiments before the formal experiment. The pre-experiment results indicated no significant differences in accuracy and response time for the same type of cognitive task across different scenes (*p* > 0.05), ensuring consistent cognitive task difficulty across scenarios.

### 2.3 Participants

By using the G*Power software, under the conditions of three levels of brightness and two levels of pattern, it was determined that at least 19 participants were needed for a repeated measures two-factor analysis of variance with within-subjects design (*α* = 0.05, power = 0.9, effect size = 0.4) to complete this study. A total of 32 undergraduate and postgraduate students were recruited as participants for this experiment, with no restrictions on their majors. 32 participants consist of 16 males (with an average age of 21.8) and 16 females (with an average age of 21.9). To prevent adverse impacts on the experimental results due to mental health conditions, participants with a history of psychiatric disorders, depression, or brain diseases were excluded. Since participants needed to wear VR glasses, which could conflict with framed glasses, only those with normal vision or corrected normal vision with contact lenses were included. All participants were instructed to maintain good sleep and mental health for a week before the experiment and to refrain from taking psychoactive drugs or stimulants. Participants reported being in good physical and mental condition before the experiment. Ethical approval was granted by the Ethics Committee of Jingdezhen Third People’s Hospital (LL2023010), in accordance with the Declaration of Helsinki. Informed consent was obtained from all participants.

### 2.4 Theory and hypotheses

To explore the perceptual and neural responses of participants to ceramic tile environments, this study adopts EEG Power Spectral Density (PSD) analysis to examine the characteristics of brain wave rhythms. The following sections elaborate on the theoretical foundations of EEG rhythms and their associations with visual comfort and cognitive performance, then propose research hypotheses based on these theories.

#### 2.4.1 Alpha waves and visual comfort

The frequency range of alpha waves is between 8 and 13 Hz and is associated with relaxation, rest, and cognitive control, typically observed in the occipital lobe (Welch, 1967). An increase in alpha waves is usually seen when individuals feel relaxed and stable, while a decrease in alpha waves may be closely linked to tension, depression, and other mental states. Previous studies have commonly associated a significant increase in alpha waves with positive environmental experiences (Choi et al., 2015). By calculating the PSD, changes in these rhythm waves can be understood. Previous studies have also confirmed that tile design features can influence individuals’ emotional states (Agost and Vergara, 2014; Laparra-Hernández et al., 2009), which may further affect alpha wave activity.

Building on the above theoretical and empirical foundations, this study proposes that alpha wave PSD will vary with the visual comfort of ceramic tile environments. Specifically:

*H*1: The α PSD will be higher in environments where participants experience greater visual comfort with ceramic tiles.

#### 2.4.2 Beta waves, visual comfort, and cognitive performance

Beta waves have a frequency range of 12 to 30 Hz. An increase in beta waves is often related to cognitive tasks, attention, thinking, and decision-making activities (Díaz et al., 2019). In studies related to electroencephalography (EEG), numerous scholars have reported that the beta band (13–30 Hz) exhibits two distinct oscillatory families, leading to its subdivision into low-frequency and high-frequency subbands (Chandrasekaran et al., 2019; Collura, 2012; Díaz et al., 2019; Gruzelier, 2014; Hoffmann, 2005; Kang et al., 2015; Lee et al., 2019; Sangal and Sangal, 2015; Smit, 2004; Wang X. et al., 2019; Wang S. Y. et al., 2019; Li J. et al., 2021; Li K. et al., 2021). Among them, Li J. et al. (2021), Li K. et al. (2021), Smit (2004), and Kang et al. (2015) et al. divided the beta band into low-frequency beta (13-22 Hz) and high-frequency beta (23-30 Hz). Some studies consider the low-frequency *β* waves to be within the healthy range of beta oscillations (Spitzer and Haegens, 2017; Díaz et al., 2019), while the increase in high-frequency beta waves is associated with stress, anxiety, thinking, and overstimulation (Engel and Fries, 2010; Abhang et al., 2016; Wang X. et al., 2019; Wang S. Y. et al., 2019). This partitioning approach has also been utilized in analogous prior research: for instance, Li J. et al. (2021), Li K. et al. (2021) employed this *β*-band subdivision when investigating cognitive performance under different wall color conditions in interior design using EEG technology. Consistent with these precedents, the beta band in the present study is subdivided into low-frequency beta (13–22 Hz) and high-frequency beta (23–30 Hz).

##### 2.4.2.1 Low-frequency beta waves and cognitive performance

In the research on the functional differences between the low-frequency and high-frequency beta wave frequency bands, Spitzer and Haegens (2017) and Díaz et al. (2019) argued that the low-frequency beta waves fall within the healthy range of beta oscillations and are involved in helping people cope with cognitive, attentional, and decision-making challenges in daily life. Some studies have found that when participants perform well in cognitive activities, there is a simultaneous increase in both low-frequency beta and *α* wave (Hanslmayr et al., 2009; Spitzer and Haegens, 2017). Given that the visual characteristics of indoor environments can affect cognitive performance (Choi et al., 2014; Plass and Kalyuga, 2019), and low-frequency beta waves are closely associated with cognitive processing, this study proposes that low-frequency beta wave PSD will vary with cognitive performance in ceramic tile environments. Specifically:

*H*2: In the environment that leads to better cognitive performance among the participants, their low-frequency β PSD will increase.

##### 2.4.2.2 High-frequency beta waves, visual comfort, and cognitive performance

Furthermore, Tallon-Baudry et al. (1998) and Smit (2004) found that high-frequency beta activity increases as individuals engage in cognitive tasks. Engel and Fries (2010), Abhang et al. (2016), Wang X. et al. (2019), and Wang S. Y. et al. (2019) suggest that the increase in high-frequency beta waves is associated with stress, anxiety, thinking, and overstimulation. Notably, the visual comfort of the environment is also closely linked to high-frequency beta wave activity. Previous studies have shown that environments with low visual comfort can induce overstimulation and mild anxiety (Kaushik et al., 2023), and such negative emotional states (overstimulation, anxiety) are precisely associated with increased high-frequency beta wave activity. Thus, there may be an indirect association between ceramic tile visual comfort and high-frequency beta wave PSD: low visual comfort may lead to overstimulation and anxiety, thereby increasing high-frequency beta wave activity.

In addition to the association with visual comfort, high-frequency beta wave activity is also closely related to cognitive performance. Smit (2004) found that the power of high-frequency beta waves was positively correlated with task accuracy and negatively correlated with reaction time, indicating that high-frequency beta wave PSD is related cognitive performance outcomes (accuracy and reaction time).

Based on the above theoretical associations between high-frequency beta waves, visual comfort, and cognitive performance, this study proposes the following two hypotheses:

*H*3: In environments with lower visual comfort, the high-frequency β PSD would be higher.

*H*4: In an environment that causes participants to have higher power of high-frequency β PSD, the accuracy rate of their tasks will be higher and the reaction time will be shorter.

### 2.5 Experimental procedure

This experiment controlled for all indoor environmental factors except for ceramic tile design. Throughout the experiment, the indoor CO2 concentration was maintained at around 400 ppm, TVOC at approximately 5 μg/m^3^, PM2.5 below 12 μg/m^3^, and PM10 below 50 μg/m^3^. The temperature was set to 23 °C with relative humidity around 60%, and proper ventilation was ensured. The lighting conditions for all the experimental materials are uniform. For the acoustic environment, noise-canceling headphones played a white noise recording from a studio to create a consistent auditory experience for participants.

**Figure 5:**
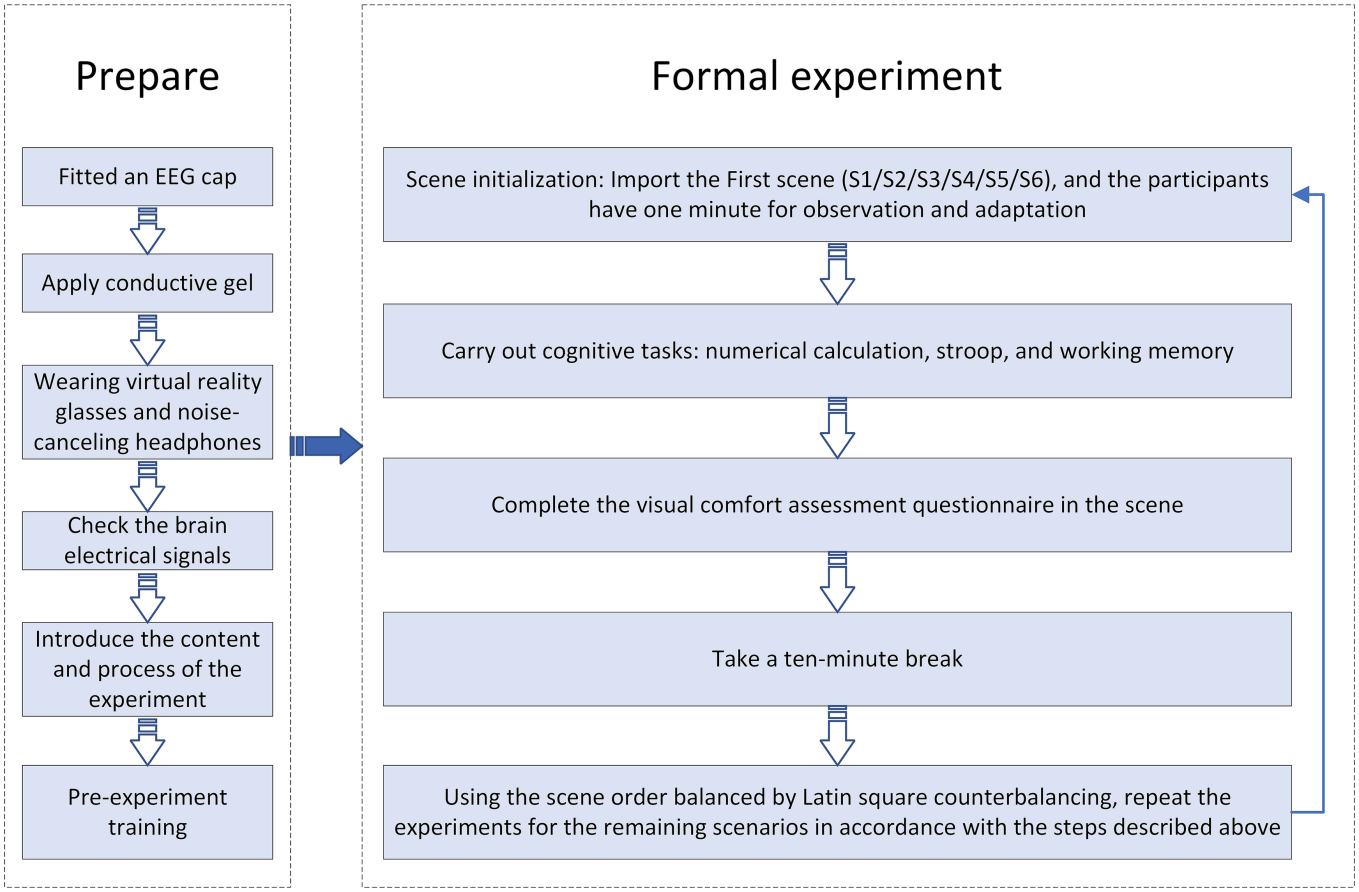
The experimental procedure.

After stabilizing the environmental factors, the experimental procedure was as follows: (1) Preparation: Participants were fitted an EEG cap, and conductive gel was applied to their scalps to maintain electrode impedance below 5 kΩ. Once the EEG cap was secured, participants wore VR goggles connected to a computer and noise-canceling headphones. (2) Signal verification: The EEG signals were examined to confirm the presence of typical eye-movement artifacts and the absence of baseline drift. (3) Brief Introduction: Briefly explain the experimental process to the participants, informing them that they will enter a virtual reality environment simulating an office. This environment is used for daily work, short-term receptions, and relaxation to help them relax and mentally prepare. (4) Pre-experiment training: A preliminary session was conducted to familiarize participants with the cognitive tasks, including numerical calculation, Stroop word-color interference, and working memory tasks, thereby minimizing the influence of task unfamiliarity on performance. (5) Scene initialization: The VR scene was loaded into Unity, and EEG recording commenced. Participants were given 1 min to observe and acclimate to the VR environment of scene 1. (6) Cognitive task execution: Following the observation period, cognitive task stimuli were presented in Unity. Participants completed three cognitive tasks while E-Prime 2.0 software recorded their responses and reaction times. Participants used the left and right mouse buttons to indicate choices presented on the left and right sides, respectively. (7) Visual comfort assessment: Upon completing the cognitive tasks, participants completed a visual comfort evaluation questionnaire in the Unity scene, rating the indoor environment from multiple perspectives while their scores were recorded. This concluded the experiment for the first scene (The experimental procedure of each scene is illustrated in Figure 5). (8) Multi-scene experimental process: The experimental process (including VR observation, cognitive tasks, and subjective questionnaires) for the 6 VR scenes (S1–S6, corresponding to different ceramic tile features) was conducted sequentially. A 10-min standardized rest interval (with VR headsets removed for full relaxation) was implemented between consecutive experimental scenes to mitigate fatigue and cybersickness, ensuring the reliability of cognitive and behavioral EEG data. The scene presentation order adopted a 6 × 6 complete Latin square design to balance order effects: 32 original participants were divided into 6 groups. The 6 groups (6 participants each) used the orders S1-S2-S3-S4-S5-S6, S2-S3-S4-S5-S6-S1, S3-S4-S5-S6-S1-S2, S4-S5-S6-S1-S2-S3, S5-S6-S1-S2-S3-S4, and S6-S1-S2-S3-S4-S5 respectively. Among the final 20 valid participants, the distribution of the scene sequence still ensured that each scene appeared multiple times in different order positions, avoiding confusion in the results due to practice or carry-over effects. Following the experimental drawing methods of similar studies (Zhao and Zhang, 2024; Zhao et al., 2026), we have depicted the detailed experimental steps in Figure 5.

**Figure 6:**
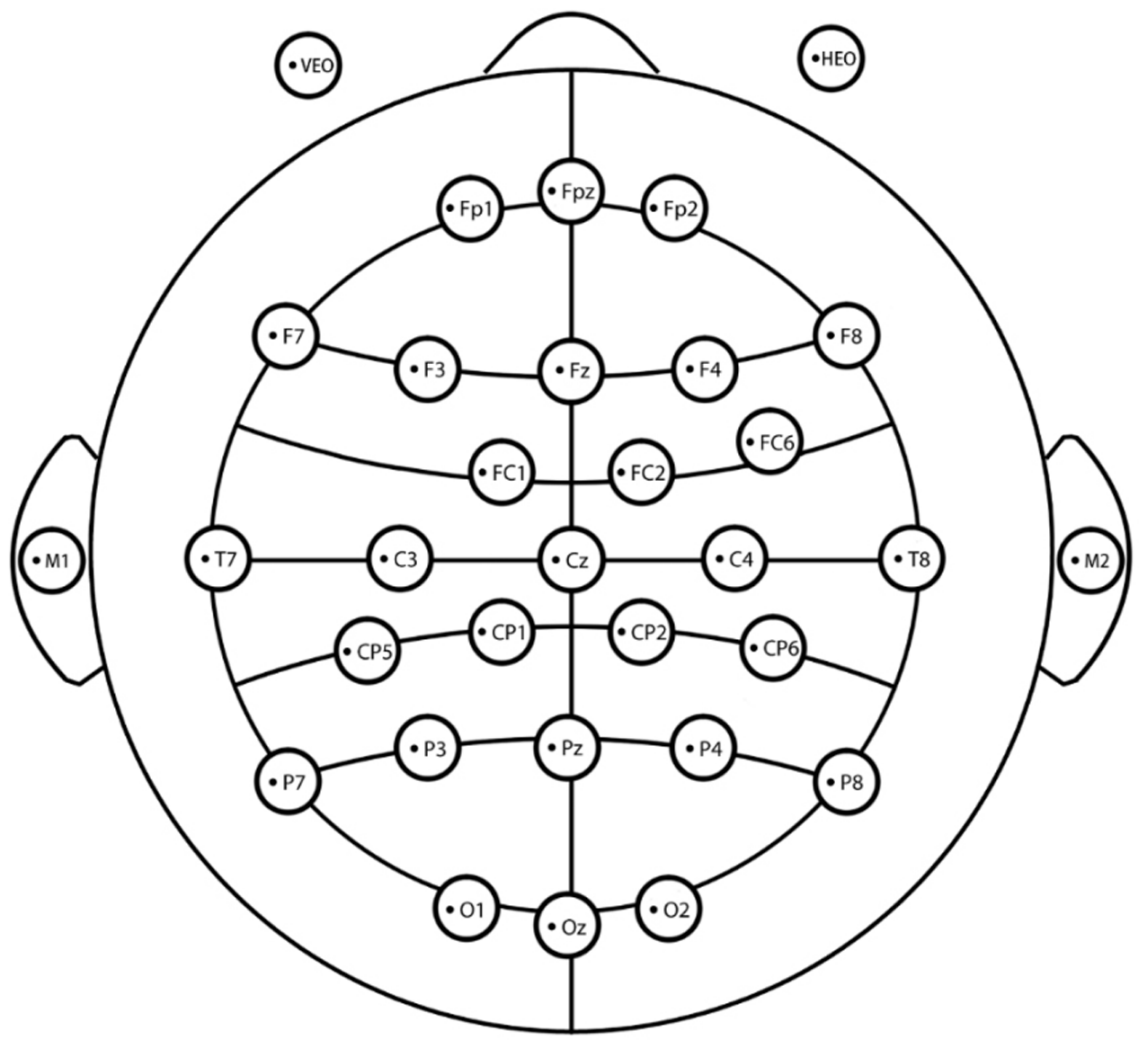
Electrode placement diagram for EEG recording.

The electrode placement is illustrated in Figure 6. Eye electrodes were checked prior to the experiment to ensure correct positioning.

Based on the hypotheses, EEG signals collected during the experiment were preprocessed using PSD analysis to extract alpha waves, low-frequency beta waves, and high-frequency beta waves under various environmental conditions. The preprocessing steps included data segmentation, electrode removal, baseline drift elimination, re-referencing, independent component analysis (ICA), fast Fourier transform (FFT), PSD extraction of alpha and beta waves, and graphical representation. The specific steps are as follows: (1) Data Segmentation: Segment the one-minute time slices after each participant begins the cognitive tasks in each of the six scenes. (2) Electrode Removal: Remove unnecessary electrodes such as HEOG and VEOG. (3) Filtering: Set the filter parameters to include frequencies above 0.5 Hz and below 30 Hz. (4) Re-referencing: Use the average of the M1 and M2 reference electrodes for re-referencing. (5) ICA: Independent Component Analysis was conducted, and ocular and muscular artifacts were manually removed. (6) FFT: Welch’s method (Welch, 1967) was applied with a baseline period of −2 to 0 s, segment length of 2 s, and 50% overlap to calculate amplitude and phase information for each frequency. (7) PSD extraction: Average PSD values for alpha, low-frequency beta, and high-frequency beta waves were computed from electrodes across environments during cognitive tasks. (8) Graphical representation: Alpha waves, associated with relaxation, and beta waves, reflecting cognitive activity, were graphically depicted.

Given the experimental design’s two-factor structure, graphical outputs focused on factor-level conditions rather than individual scenes. For instance, environments featuring patterned tiles with varying brightness levels were grouped under “patterned conditions.” Therefore, topographic and power spectral maps were produced separately for pattern and brightness factors, analyzing frequency bands of 8–13 Hz, 14–22 Hz, and 23–30 Hz.

ANOVA was used for data analysis, with statistical significance set at *p* < 0.05. Before analysis, normality and homogeneity of variance tests were conducted. This experiment follows a two-level pattern factor crossed with a three-level brightness factor design. Independent variables included tile pattern and brightness, while dependent variables encompassed reaction time, cognitive accuracy, and PSD of alpha and beta waves. Reaction times, cognitive accuracy, and the PSD of alpha and beta waves from all six scenes were input into a two-way ANOVA. Initial analyses revealed significant main effects of electrode position on alpha waves, low-frequency beta waves, and high-frequency beta waves. To clearly present the results, we report the impacts of pattern and brightness factors on alpha waves, low-frequency beta waves, and high-frequency beta waves for each electrode region. In addition, correlations between visual comfort, cognitive performance, and EEG metrics (PSD of alpha and beta waves) were also reported.

## 3 Results

This study aimed to explore visual comfort and cognitive performance within different indoor tile design environments and to establish relevant evaluation methods. After excluding 12 datasets due to incomplete or abnormal signals from an initial pool of 32, 20 valid datasets remained for analysis. The 20 valid dataset consists of the data from 9 males (with an average age of 21.6) and 11 females (with an average age of 21.7). According to previous literature, Power (1-*β*) should be higher than 0.8, and 0.9 is a relatively high power (Jones et al., 2003; Lan and Lian, 2010). The valid data retained in this article amounts to 20, which is more than the minimum sample size required when the statistical power is 0.9. Therefore, the remained data can still be used for statistical analysis. Visual comfort was assessed through subjective evaluations and alpha wave PSD, while cognitive performance was evaluated via cognitive task outcomes and beta wave PSD. The following sections detail results concerning cognitive performance, subjective evaluations, and the PSD analysis of alpha, low-frequency beta, and high-frequency beta waves.

### 3.1 Cognitive reaction time

Participants’ cognitive performance was primarily measured using reaction time and accuracy.

For reaction time, tile pattern and brightness both have significant main effects on participants’ cognitive reaction time, while their interaction effect is not significant, with shorter reaction times observed in patterned and light-toned tile environments. For specifically, tile pattern and brightness both had significant main effects [pattern: *F* (1, 19) = 4.436, *p* = 0.049, η^2^ = 0.189; brightness: *F* (2, 38) = 6.278, *p* = 0.006, η^2^ = 0.248], while their interaction effect was not significant [F (2, 38) = 0.636, *p* = 0.504, η^2^ = 0.032]. Participants had shorter reaction times in environments with patterned tiles (mean = 3852.62 ms), and the shortest reaction times were observed in environments with light-toned tiles (mean = 3819.75 ms). These results suggest that tile pattern and brightness can significantly affect the speed of cognitive processing, providing initial evidence for the impact of tile visual characteristics on cognitive performance. Detailed results are presented in Appendix Tables 1, 2.

### 3.2 Cognitive accuracy rate

Neither tile pattern nor brightness has a significant main effect on cognitive accuracy, and their interaction effect is also not significant. A repeated-measures two-factor ANOVA showed that neither tile pattern nor brightness had a significant main effect on cognitive accuracy, and their interaction effect was also not significant [pattern: F (1, 19) = 0.172, *p* = 0.683, η^2^ = 0.009; brightness: F (2, 38) = 0.188, *p* = 0.807, η^2^ = 0.01; interaction: F (2, 38) = 0.96, *p* = 0.386, η^2^ = 0.048], indicating that tile characteristics did not affect cognitive accuracy. This finding indicates that although tile visual characteristics influence cognitive processing speed (as shown in Section 3.1), they do not affect the accuracy of cognitive tasks. Detailed results are shown in Appendix Tables 3, 4.

### 3.3 Visual comfort score

Both tile pattern and brightness have significant main effects on participants’ visual comfort scores, with no significant interaction effect, and higher comfort scores are observed in non-patterned and light-toned tile environments. The reliability of the visual comfort evaluation questionnaire (Chen et al., 2024) was verified by Cronbach’s alpha coefficient (*α* = 0.90), indicating high reliability. ANOVA results showed that both tile pattern and brightness had significant main effects on visual comfort scores [pattern: *F* (1, 19) = 6.786, *p* = 0.017, η^2^ = 0.263; brightness: *F* (2, 38) = 34.22, *p* < 0.001, η^2^ = 0.643], with no significant interaction effect: [F (2, 38) = 2.128, *p* = 0.144, η^2^ = 0.101]. Specifically, participants gave significantly higher visual comfort scores to non-patterned tile environments (M = 3.29) and light-toned tile environments (M = 4.031), respectively. These results confirm that non-patterned and light-toned tiles are more likely to bring positive subjective visual comfort experiences to participants. Detailed data are in Appendix Tables 5, 6.

### 3.4 The power spectral density of alpha and beta waves

**Figure 7:**
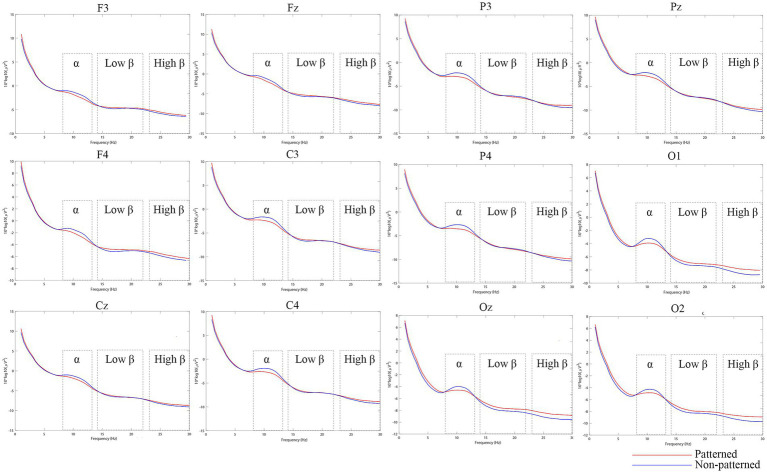
Power spectra of frontal, central, parietal, and occipital regions induced by two levels of pattern factors.

**Figure 8:**
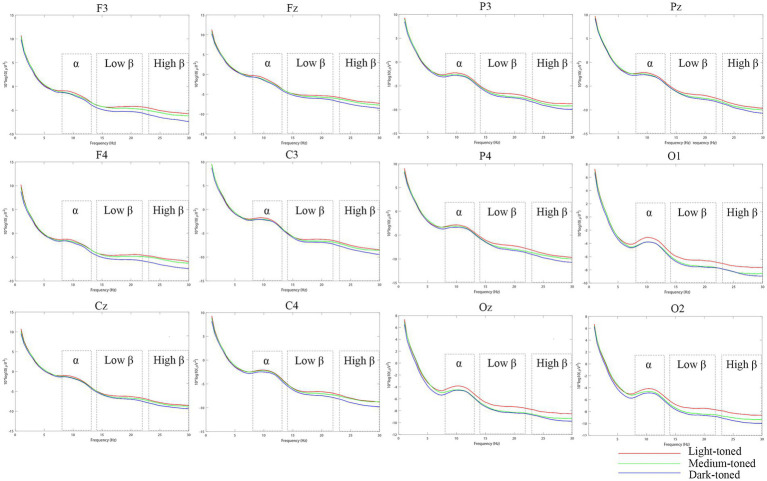
Power spectra of the frontal, central, parietal, and occipital regions induced by three levels of brightness factor.

**Figure 9:**
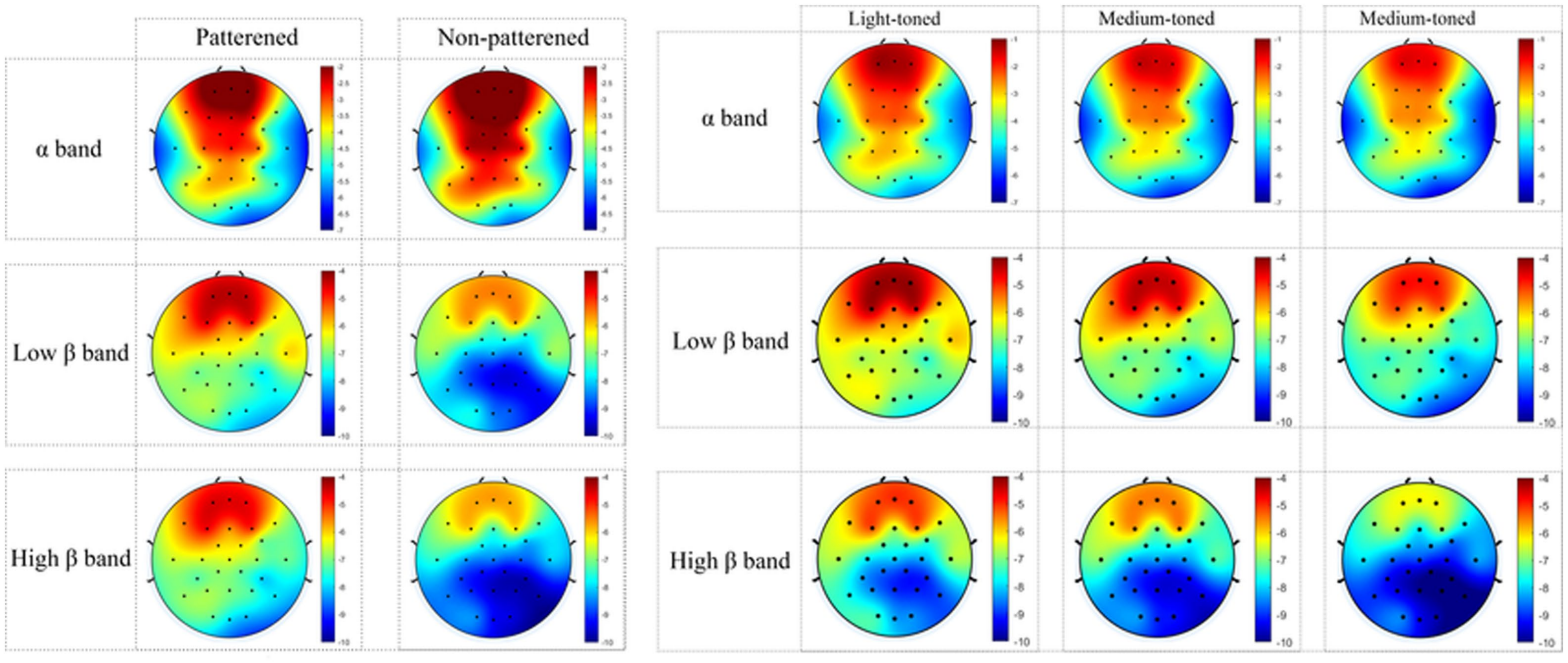
Brain topographic maps of alpha, low frequency beta, and high frequency beta waves induced by pattern and brightness factors at two levels [10*log_10_(μV^2^/Hz)].

Figures 7–9 show the alpha and beta wave PSD spectra and brain topographic maps under different tile pattern and brightness conditions (see figure captions for details). In Figure 7, the frequency range of the power spectra is 0–30 Hz. In the figures, the red lines represent the power spectra in the patterned tile environments, while the blue lines represent the power spectra in the non-patterned tile environments. In Figure 8, the red lines represent the power spectra in the light-toned tile environment, the green lines represent the power spectra in the medium-toned tile environment, and the blue lines represent the power spectra in the dark-toned tile environment. Figure 9 depict brain topographic maps for alpha, low-frequency beta, and high-frequency beta waves as influenced by pattern and brightness factors.

#### 3.4.1 Effects of pattern and brightness on alpha wave power spectral density

Tile pattern has a significant effect on alpha wave PSD in the frontal, central, and parietal regions, while brightness only significantly affects alpha PSD in the frontal region, with no significant interaction effects between pattern and brightness in any of these regions. Specifically, in the frontal region, both pattern and brightness had significant main effects [pattern: *F* (1, 19) = 6.774, *p* = 0.017, η^2^ = 0.263; brightness: *F* (2, 38) = 4.865, *p* = 0.014, η^2^ = 0.204] with no significant interaction, and alpha PSD was higher in non-patterned (M = −1.622) and light-toned tile environments (mean = −1.621). In the central region, only pattern had a significant main effect [F (1, 19) = 7.850, *p* = 0.011, η^2^ = 0.292], with higher alpha PSD in non-patterned (mean = −1.9) and light-toned (mean = −2.029) tiles. In the parietal region, pattern also had a significant main effect [F (1, 19) = 6.445, *p* = 0.020, η^2^ = 0.253], with higher alpha PSD in non-patterned environments (mean = −2.589). Additionally, Pearson correlation analysis showed a significant positive correlation between frontal alpha PSD and visual comfort score (*p* = 0.02, *r* = 0.514). These results indicate that alpha wave PSD is closely associated with visual comfort, especially in the frontal region, which supports the use of alpha wave PSD as a physiological indicator of visual comfort. Detailed results are presented in Appendix Tables 7–10.

#### 3.4.2 Effects of pattern and brightness on low-frequency and high-frequency beta wave power spectral density

Brightness has a significant main effect on both low-frequency and high-frequency beta wave PSD in all four brain regions, while pattern only significantly affects beta PSD in the occipital region, with no significant interaction effects between the two factors. In the frontal, central, and parietal regions, brightness was the dominant factor, with the highest low-frequency and high-frequency beta PSD observed in light-toned tile environments (e.g., frontal low-frequency: mean = −4.542; central high-frequency: mean = −8.039), while pattern had no significant effect. In the occipital region, both pattern [low-frequency: F (1, 19) = 5.092, *p* = 0.036, η^2^ = 0.211; high-frequency: F (1, 19) = 8.381, *p* = 0.009, η^2^ = 0.306] and brightness [low-frequency: F (2, 38) = 13.556, *p* < 0.001, η^2^ = 0.416; high-frequency: F (2, 38) = 8.524, *p* = 0.001, η^2^ = 0.31] had significant main effects, with higher beta PSD in patterned and light-toned tile environments. Correlation analysis showed a negative correlation between beta wave PSD and reaction time (*p* = 0.037, *r* = −0.468; *p* = 0.045, *r* = −0.453), but no correlation with cognitive accuracy. These results confirm that beta wave PSD (especially low-frequency beta) is closely related to cognitive performance, and the occipital region is more sensitive to tile pattern, which is consistent with the functional characteristics of the occipital lobe in visual processing. Detailed data are presented in Appendix Tables 11–18.

### 3.5 Overall conclusion

In the terms of visual comfort, the results indicate that young university students experienced higher visual comfort in environments with non-patterned and light-toned ceramic tiles, and have higher alpha PSD associated with positive experiences.

In the terms of cognitive Performance, Cognitive experiments showed that young university students had shorter reaction times in environments with patterned and light-toned ceramic tiles compared to other settings. In patterned tile environments, participants exhibited lower alpha waves and higher beta waves, indicating focused attention and some tension. In the light-toned tile environment, the higher alpha wave PSD value indicates relaxation, while a higher beta wave indicates alertness and attention during cognitive tasks. This suggests that people are in a state of relaxation yet focused in this environment.

## 4 Discussion

The visual comfort evaluation results indicate that young university students rated environments with non-patterned and light-toned ceramic tiles higher in visual comfort, while environments with patterned and dark-toned tiles received lower ratings. According to the questionnaire results across various dimensions, this may be because non-patterned and light-toned tile environments appear cleaner, more spacious, and appropriately bright, leading to pleasant and relaxing visual comfort. This finding corroborates previous questionnaire survey results (Chen et al., 2024).

The analysis of alpha wave PSD showed a significant increase in alpha wave PSD in environments with non-patterned and light-toned ceramic tiles. Prior research indicates that increased alpha wave PSD in the occipital region reflects relaxation and reduced anxiety during restful states (Moini and Piran, 2020). The correlation between subjective visual comfort ratings and alpha wave PSD confirms, from both psychological and neural perspectives, that individuals feel more relaxed and comfortable in non-patterned and light-toned tile environments. Therefore, Hypothesis 1 was verified. These findings provide physiological evidence supporting previous assessments of pattern and brightness in visual comfort.

Cognitive performance results revealed that young university students had shorter cognitive reaction times in environments with patterned and light-toned ceramic tiles, where beta wave PSD analysis also indicated higher activity. Therefore, Hypothesis 2 was confirmed. Urthermore, compared to the data in the environment with light-colored tiles, in the environment with dark-colored tiles that cause lower visual comfort, the frequency of the high-frequency beta waves generated by people is lower. This indicates that Hypothesis 3 does not hold true. On the other hand, in the environment with light-toned ceramic tiles that generate higher high-frequency beta PSD, people’s reaction times were shorter, while there was no significant difference in accuracy. Therefore, Hypothesis 4 was basically supported. In this study, the low-frequency and high-frequency beta waves in each condition seemed to increase or decrease simultaneously. That is, the same conditions did not lead to a difference between the low-frequency and high-frequency beta waves. Therefore, in the subsequent discussion, these two sub-frequency bands of the beta waves will be collectively referred to as beta waves for discussion. Kamiński et al. noted that beta waves are associated with alertness and focused attention, often observed during cognitive tasks (Kamiński et al., 2012). Increased beta wave PSD may indicate anxiety and stress (Ribas et al., 2018) as well as heightened focused attention. A moderate elevation in beta wave activity has been found to enhance attention and cognitive performance (Collell and Fauquet, 2015; Li et al., 2020). From an environmental psychology perspective (Zhan, 1988), reduced brightness contrast between tasks and their background can lower visual strain and glare, improving cognitive performance. The faster cognitive reactions in light-toned tile environments may be due to this reason. Additionally, the improved cognitive performance in patterned tile environments could be linked to the physiological arousal associated with higher beta wave activity (Tian et al., 2021). Higher arousal in light-toned and patterned tile environments may lead to better attention and shorter reaction times. The observed correlation between reaction times and beta wave activity further supports the relationship between beta wave activity and cognitive performance (Kamiński et al., 2012; Li et al., 2020). Combining these findings, this study suggests that the increase in alpha and overall beta waves, along with reduced cognitive reaction times in light-toned tile environments, indicates a state of relaxed yet focused attention. In patterned tile environments, the decrease in alpha waves and the increase in beta waves and cognitive performance may imply that the environment induces some stress and tension, which enhances attention.

From the theoretical point of view, this study established physiological measurement methods for assessing the visual comfort of indoor ceramic tile designs and evaluating their impact on cognitive performance. VR experiments combined with EEG measures validated subjective questionnaire outcomes from previous study (Chen et al., 2024), with increased alpha wave activity further corroborating visual comfort assessments. Alpha wave PSD thus offers a physiological measure for visual comfort evaluations in ceramic tile design. In addition, cognitive experiments conducted in VR environments evaluated the cognitive performance of young college students under different tile environments, and discovered the changes of beta wave PSD under different tile characteristics, expanding the scope and content of indoor tile visual perception research.

From the practical point of view, designers can use the findings from this study to select ceramic tiles that meet specific needs of different indoor environments. For instance, tiles that enhance alpha waves can be used in relaxing spaces such as lounges and bedrooms. Tiles that moderately increase beta waves can be selected for workspaces, meeting rooms, and offices to reduce cognitive response time. Although most participants had similar subjective evaluations and neural responses, there were some individual differences. Therefore, designers and researchers should consider the specific experiences and needs of individual users when planning designs. It should be noted that regulatory constraints on ceramic tile application are scenario-specific—cross-national regulatory differences are minimal in dry indoor environments, while regulatory requirements for high-traffic public areas may lead to divergence in application ratios (The tiles used in this article are more likely to be in line with the general situation in China.), though this is not the core research scope of this paper. This article focuses on the impact of different characteristics of tiles on visual comfort, cognitive performance, and neural responses. In addition, the actual factors influencing the design of office floors also include slip resistance, wear resistance, compliance with national building regulations, and long-term maintenance costs, which are also important factors beyond visual characteristics. For example, in slippery areas, tiles with a higher friction coefficient should be selected.

## 5 Limitation

Due to the experiments in VR experiments takes a long time, including too many factors can lead to participant fatigue, this study focused on evaluating two key variables of ceramic tile design. The coordination between ceramic tiles and other interior design (Walls, window frames and furniture, etc.) and participant group factors may also influence people’s evaluations of visual comfort and cognitive performance, and the authors will incorporate more factors for exploration in future research. In addition, sample bias may exist because all participants were university students rather than general office workers. Although this sample ensures homogeneity in cognitive ability and learning background, it may limit the generalizability of the findings to real occupational populations. The task deviation stems from the selection of the three types of cognitive tasks that are commonly used in cognitive reaction research (numerical calculation, Stroop task, and numerical memory). These tasks reflect selective attention, impulse control, and working memory but cannot cover all aspects of daily work cognition. These biases do not invalidate the conclusions but indicate boundaries of the present findings. Future studies can adopt more diverse samples and comprehensive cognitive measures to enhance generalizability.

## Data Availability

The original contributions presented in the study are included in the article/supplementary material, further inquiries can be directed to the corresponding author.
