# Supplementary material for: The influence of ceramic tile characteristics on visual comfort and cognitive performance in workplace environments: a study based on VR and EEG
Source: Front Psychol. 2026 Mar 17;17:1639942. doi: 10.3389/fpsyg.2026.1639942 (PMC13036855; doi:10.3389/fpsyg.2026.1639942)
Supplement: Supplementary file 1 [file Supplementary_file_1.docx]

**Appendices**

**Table 1.** ANOVA Results for Participants' Response Times (ms) in Environments with Different Tile Pattern levels.

| Variable | Patterned | | Non-texured | | F | P | η^2^ |
| --- | --- | --- | --- | --- | --- | --- | --- |
|  | Mean | SD | Mean | SD |  |  |  |
| Response time | 3852.62 | 122.18 | 4127.31 | 126.32 | 4.436 | 0.049^*^ | 0.189 |

**Table 2.** ANOVA Results for Participants' Response Times (ms) in Environments with Tiles of Different Brightness Levels.

| Variable | Light-toned | | Medium-toned | | Dark-toned | | F | P | η^2^ |
| --- | --- | --- | --- | --- | --- | --- | --- | --- | --- |
|  | Mean | SD | Mean | SD | Mean | SD |  |  |  |
| Response time | 3819.75 | 102.17 | 3912.47 | 147.18 | 4237.67 | 146.61 | 6.278 | 0.006^*^ | 0.248 |

**Table 3.** ANOVA Results for Participants' Cognitive Accuracy Rates (%) in Environments with Different Tile Pattern levels.

| Variable | Patterned | | Non-patterned | | F | P | η^2^ |
| --- | --- | --- | --- | --- | --- | --- | --- |
|  | Mean | SD | Mean | SD |  |  |  |
| Accuracy | 77.2% | 0.018 | 76.3% | 0.018 | 0.172 | 0.683 | 0.009 |

**Table 4.** ANOVA Results for Participants' Cognitive Accuracy Rates (%) in Environments with Tiles of Different Brightness Levels.

| Variable | Light-toned | | Medium-toned | | Dark-toned | | F | P | η^2^ |
| --- | --- | --- | --- | --- | --- | --- | --- | --- | --- |
|  | Mean | SD | Mean | SD | Mean | SD |  |  |  |
| Accuracy | 77.8% | 0.022 | 76.7% | 0.025 | 75.8% | 0.023 | 0.188 | 0.807 | 0.01 |

**Table 5.** ANOVA Results for Participants' Visual Comfort Scores with Different Tile Pattern levels.

| Variable | Patterned | | Non-patterned | | F | P | η^2^ |
| --- | --- | --- | --- | --- | --- | --- | --- |
|  | Mean | SD | Mean | SD |  |  |  |
| Visual comfort | 2.992 | 0.135 | 3.29 | 0.087 | 6.786 | 0.017^*^ | 0.263 |

**Table 6.** ANOVA Results for Participants' Visual Comfort Scores in Environments with Tiles of Different Brightness Levels.

| Variable | Light-toned | | Medium-toned | | Dark-toned | | F | P | η^2^ |
| --- | --- | --- | --- | --- | --- | --- | --- | --- | --- |
|  | Mean | SD | Mean | SD | Mean | SD |  |  |  |
| Visual comfort | 4.031 | 0.122 | 2.95 | 0.144 | 2.441 | 0.178 | 34.22 | < 0.001^*^ | 0.643 |

**Table 7.** ANOVA Results for Alpha Wave PSD [10×log₁₀(μV²/Hz)] in the Frontal Region by Pattern and Brightness Factors.

| Variable | Main effect | | | Level | Mean | SD |
| --- | --- | --- | --- | --- | --- | --- |
|  | F | P | η^2^ |  |  |  |
| Pattern | 6.774 | 0.017^*^ | 0.263 | Patterned | -2.166 | 0.495 |
|  |  |  |  | Non-patterned | -1.622 | 0.542 |
| Brightness | 4.865 | 0.014^*^ | 0.204 | Light-toned | -1.621 | 0.531 |
|  |  |  |  | Medium-toned | -1.912 | 0.502 |
|  |  |  |  | Dark-toned | -2.149 | 0.521 |

**Table 8.** ANOVA Results for Alpha Wave PSD [10×log₁₀(μV²/Hz)] in the Central Region by Pattern and Brightness Factors.

| Variable | Main effect | | | Level | Mean | SD |
| --- | --- | --- | --- | --- | --- | --- |
|  | F | P | η^2^ |  |  |  |
| Pattern | 7.85 | 0.011^*^ | 0.292 | Patterned | -2.574 | 0.575 |
|  |  |  |  | Non-patterned | -1.9 | 0.644 |
| Brightness | 2.452 | 0.106 | 0.114 | Light-toned | -2.029 | 0.607 |
|  |  |  |  | Medium-toned | -2.255 | 0.593 |
|  |  |  |  | Dark-toned | -2.428 | 0.623 |

**Table 9.** ANOVA Results for Alpha Wave PSD [10×log₁₀(μV²/Hz)] in the Parietal Region by Pattern and Brightness Factors.

| Variable | Main effect | | | Level | Mean | SD |
| --- | --- | --- | --- | --- | --- | --- |
|  | F | P | η^2^ |  |  |  |
| Pattern | 6.445 | 0.02^*^ | 0.253 | Patterned | -3.318 | 0.631 |
|  |  |  |  | Non-patterned | -2.589 | 0.742 |
| Brightness | 2.292 | 0.125 | 0.108 | Light-toned | -2.689 | 0.676 |
|  |  |  |  | Medium-toned | -3 | 0.679 |
|  |  |  |  | Dark-toned | -3.171 | 0.705 |

**Table 10.** ANOVA Results for Alpha Wave PSD [10×log₁₀(μV²/Hz)] in the Occipital Region by Pattern and Brightness Factors.

| Variable | Main effect | | | Level | Mean | SD |
| --- | --- | --- | --- | --- | --- | --- |
|  | F | P | η^2^ |  |  |  |
| Pattern | 3.39 | 0.081 | 0.151 | Patterned | -4.61 | 0.912 |
|  |  |  |  | Non-patterned | -4.1 | 0.953 |
| Brightness | 5.605 | 0.011^*^ | 0.228 | Light-toned | -3.928 | 0.967 |
|  |  |  |  | Medium-toned | -4.507 | 0.908 |
|  |  |  |  | Dark-toned | -4.63 | 0.918 |

**Table 11.** ANOVA Results for Low-Frequency Beta Wave PSD [10×log₁₀(μV²/Hz)] in the Frontal Region by Pattern and Brightness Factors.

| Variable | Main effect | | | Level | Mean | SD |
| --- | --- | --- | --- | --- | --- | --- |
|  | F | P | η^2^ |  |  |  |
| Pattern | 0.666 | 0.424 | 0.034 | Patterned | -4.931 | 0.597 |
|  |  |  |  | Non-patterned | -5.045 | 0.601 |
| Brightness | 13.762 | <0.001^*^ | 0.42 | Light-toned | -4.542 | 0.664 |
|  |  |  |  | Medium-toned | -4.941 | 0.603 |
|  |  |  |  | Dark-toned | -5.48 | 0.539 |

**Table 12.** ANOVA Results for High-Frequency Beta Wave PSD [10×log₁₀(μV²/Hz)] in the Frontal Region by Pattern and Brightness Factors.

| Variable | Main effect | | | Level | Mean | SD |
| --- | --- | --- | --- | --- | --- | --- |
|  | F | P | η^2^ |  |  |  |
| Pattern | 1.381 | 0.255 | 0.068 | Patterned | -6.19 | 0.715 |
|  |  |  |  | Non-patterned | -6.456 | 0.72 |
| Brightness | 18.725 | <0.001^*^ | 0.496 | Light-toned | -5.66 | 0.794 |
|  |  |  |  | Medium-toned | -6.165 | 0.712 |
|  |  |  |  | Dark-toned | -7.143 | 0.655 |

**Table 13.** ANOVA Results for Low-Frequency Beta Wave PSD [10×log₁₀(μV²/Hz)] in the Central Region by Pattern and Brightness Factors.

| Variable | Main effect | | | Level | Mean | SD |
| --- | --- | --- | --- | --- | --- | --- |
|  | F | P | η^2^ |  |  |  |
| Pattern | 0.361 | 0.555 | 0.019 | Patterned | -6.514 | 0.543 |
|  |  |  |  | Non-patterned | -6.603 | 0.565 |
| Brightness | 6.842 | 0.005^*^ | 0.265 | Light-toned | -6.279 | 0.58 |
|  |  |  |  | Medium-toned | -6.563 | 0.572 |
|  |  |  |  | Dark-toned | -6.833 | 0.513 |

**Table 14.** ANOVA Results for High-Frequency Beta Wave PSD [10×log₁₀(μV²/Hz)] in the Central Region by Pattern and Brightness Factors.

| Variable | Main effect | | | Level | Mean | SD |
| --- | --- | --- | --- | --- | --- | --- |
|  | F | P | η^2^ |  |  |  |
| Pattern | 2.206 | 0.154 | 0.104 | Patterned | -8.268 | 0.657 |
|  |  |  |  | Non-patterned | -8.54 | 0.646 |
| Brightness | 10.044 | <0.001^*^ | 0.346 | Light-toned | -8.039 | 0.696 |
|  |  |  |  | Medium-toned | -8.247 | 0.668 |
|  |  |  |  | Dark-toned | -8.926 | 0.601 |

**Table 15.** ANOVA Results for Low-Frequency Beta Wave PSD [10×log₁₀(μV²/Hz)] in the Parietal Region by Pattern and Brightness Factors.

| Variable | Main effect | | | Level | Mean | SD |
| --- | --- | --- | --- | --- | --- | --- |
|  | F | P | η^2^ |  |  |  |
| Pattern | 0.029 | 0.866 | 0.002 | Patterned | -7.081 | 0.58 |
|  |  |  |  | Non-patterned | -7.054 | 0.597 |
| Brightness | 12.172 | <0.001^*^ | 0.39 | Light-toned | -6.674 | 0.6 |
|  |  |  |  | Medium-toned | -7.165 | 0.6 |
|  |  |  |  | Dark-toned | -7.363 | 0.568 |

**Table 16.** ANOVA Results for High-Frequency Beta Wave PSD [10×log₁₀(μV²/Hz)] in the Parietal Region by Pattern and Brightness Factors.

| Variable | Main effect | | | Level | Mean | SD |
| --- | --- | --- | --- | --- | --- | --- |
|  | F | P | η^2^ |  |  |  |
| Pattern | 2.58 | 0.125 | 0.12 | Patterned | -9.186 | 0.677 |
|  |  |  |  | Non-patterned | -9.454 | 0.649 |
| Brightness | 16.338 | <0.001^*^ | 0.462 | Light-toned | -8.874 | 0.692 |
|  |  |  |  | Medium-toned | -9.273 | 0.68 |
|  |  |  |  | Dark-toned | -9.812 | 0.62 |

**Table 17.** ANOVA Results for Low-Frequency Beta Wave PSD [10×log₁₀(μV²/Hz)] in the Occipital Region by Pattern and Brightness Factors.

| Variable | Main effect | | | Level | Mean | SD |
| --- | --- | --- | --- | --- | --- | --- |
|  | F | P | η^2^ |  |  |  |
| Pattern | 5.092 | 0.036^*^ | 0.211 | Patterned | -7.361 | 0.907 |
|  |  |  |  | Non-patterned | -7.741 | 0.889 |
| Brightness | 13.556 | <0.001^*^ | 0.416 | Light-toned | -6.947 | 0.942 |
|  |  |  |  | Medium-toned | -7.767 | 0.884 |
|  |  |  |  | Dark-toned | -7.937 | 0.878 |

**Table 18.** ANOVA Results for High-frequency Beta Wave PSD [10×log₁₀(μV²/Hz)] in the Occipital Region by Pattern and Brightness Factors.

| Variable | Main effect | | | Level | Mean | SD |
| --- | --- | --- | --- | --- | --- | --- |
|  | F | P | η^2^ |  |  |  |
| Pattern | 8.381 | 0.009^*^ | 0.306 | patterned | -8.377 | 1.012 |
|  |  |  |  | Non-patterned | -9.066 | 0.962 |
| Brightness | 8.524 | 0.001^*^ | 0.31 | Light-toned | -8.071 | 1.042 |
|  |  |  |  | Medium-toned | -8.887 | 0.96 |
|  |  |  |  | Dark-toned | -9.206 | 0.975 |
